# *Pneumocystis jirovecii* pneumonia in a patient with *Mycobacterium intracellulare* pulmonary disease: a case report

**DOI:** 10.1186/s12879-025-12165-x

**Published:** 2025-11-24

**Authors:** Xiaoni Zhou, Xinhua Xiao, Yuying Wu, Jun Wang, Zhiyun Pan, Han Wang, Xiaohui Luo, Zhi Yao

**Affiliations:** Department of Pulmonary and Critical Care Medicine, Wuhan Pulmonary Hospital (Wuhan Institute for Tuberculosis Control), No.28 Baofeng Road, Wuhan, Hubei 430030 China

**Keywords:** Nontuberculous mycobacteria, *Pneumocystis jirovecii* pneumonia, Immunocompromised

## Abstract

This report details the case of a 77-year-old female with *Mycobacterium intracellulare* pulmonary disease and history of bladder tumor surgery. The patient initially presented to our hospital with a one-day history of fever. After receiving antibiotic and anti-nontuberculous mycobacterial therapy for three days, the patient remained febrile. The patient’s white blood cell and neutrophil counts were within normal range, while the lymphocyte count was decreased. Chest computed tomography (CT) revealed diffuse ground-glass opacities in the lungs. Further investigations demonstrated a significantly reduced CD4 + T-lymphocyte count. *Mycobacterium intracellulare* and *Pneumocystis jirovecii* (PJ) were identified in the bronchoalveolar lavage fluid (BALF) specimen. The patient was treated with oral trimethoprim-sulfamethoxazole (TMP-SMZ) and intravenous caspofungin. Following treatment, the patient’s symptoms improved, and she was discharged. This study reports the first documented case of rare *Pneumocystis jirovecii* pneumonia (PJP) complicating nontuberculous mycobacterial lung disease. It underscores the importance of monitoring immune function and facilitating the early recognition of secondary infections in such patients.

## Background

Nontuberculous mycobacteria (NTM), defined as mycobacterial species other than the *Mycobacterium tuberculosis* complex and *Mycobacterium leprae*, are ubiquitous in the natural environment, particularly in water and soil. These organisms can colonize and infect various organs and tissues in humans, with NTM pulmonary disease being the most common clinical manifestation. The *Mycobacterium avium* complex (MAC) is one of the most frequently encountered groups of NTM. These organisms are slow-growing mycobacteria and include representative species such as *Mycobacterium avium*, *Mycobacterium intracellulare*, *Mycobacterium chimaera*, and *Mycobacterium marseillense* [1–4]. PJP is a fungal infection of the respiratory system caused by PJ. It predominantly affects immunocompromised or immunodeficient individuals and represents the most common opportunistic respiratory infection in patients with acquired immunodeficiency syndrome [5]. However, in recent years, the proportion of non-HIV-associated PJP cases has increased significantly, with a notably higher mortality rate compared to HIV-infected populations [6]. Diagnostic delay is a major contributor to poor prognosis in non-HIV-associated PJP [7]. Therefore, we specifically describe a rare case of MAC co-infection with PJP to enhance clinical awareness and aid in preventing misdiagnosis and delayed diagnosis.

## Case presentation

**Fig. 1:**
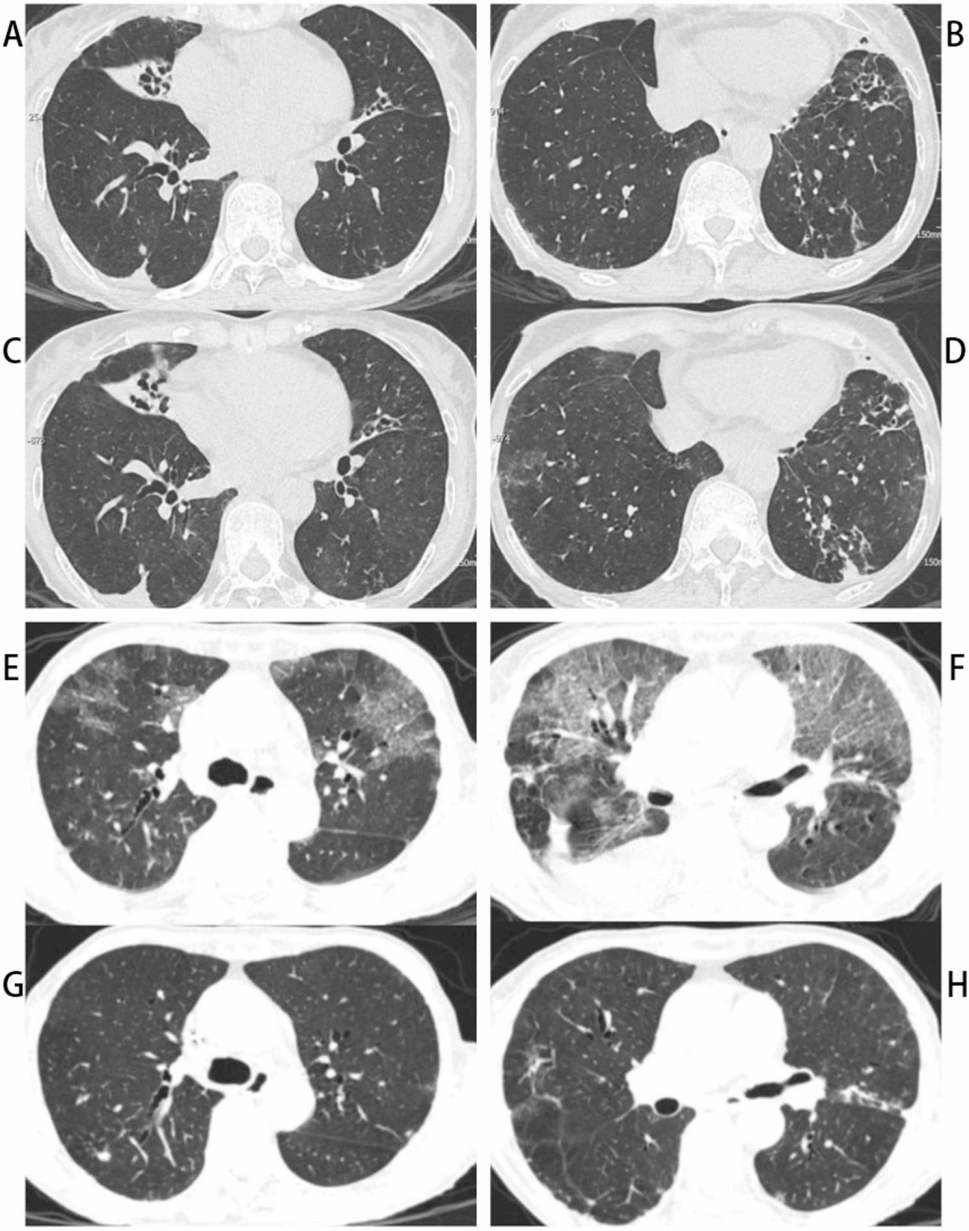
Chest CT scans of the patient. (**A, B**) Pulmonary window of lung CT on June 17, 2024. (**C**, **D**) Lung CT scan on August 15 showed new patchy opacities emerged in the left lower lobe. (**E**, **F**) Lung CT scan showed diffuse bilateral ground-glass opacities with interlobular and intralobular septal thickening, accompanied by bilateral pleural effusions on August 26. (**G**, **H**) CT scan revealed the regression of the changes in the lungs after treatments on September 6, 2024

A 77-year-old female patient who presented with a 1-day history of hyperpyrexia, was admitted to our respiratory department on August 15, 2024. The patient underwent surgery for bladder cancer in 2004 and was diagnosed with coronary heart disease in 2008. In 2019, a diagnosis of bronchiectasis complicated by NTM pulmonary disease was established. On April 3, 2019, the patient’s bronchoalveolar lavagefluid (BALF) culture was positive for nontuberculous mycobacteria, subsequently identified as *Mycobacterium intracellulare*. According to the British Thoracic Society Guideline, she was prescribed an oral NTM regimen consisting of azithromycin, isoniazid over the following two years, rifampicin and ethambutol. However, her medication adherence was poor and took her medications irregularly. Follow-up BALF cultures in August 2020 and May 2021 remained positive for NTM, despite concurrent negative sputum cultures. All antimycobacterial therapy was discontinued after May 2021. Physical examination on admission revealed a temperature of 36.5 °C, a heart rate of 78 bpm, a respiratory rate of 20/min, a blood pressure of 98/55 mmHg, and an oxygen saturation of 100% without supplemental oxygen. Both lungs had clear respiratory sounds and no rales. Auxiliary examinations were performed: white blood cell count 7.68 × 10⁹/L, neutrophils 6.15 × 109/L, lymphocytes 0.68 × 109/L, hemoglobin 106 g/L (Table 1); albumin 32.5 g/L, aspartate aminotransferase 50 U/L; high-sensitivity C-reactive protein 21.45 mg/L, interleukin-6 25.17 pg/mL, procalcitonin within normal range; serology: negative for hepatitis, syphilis, and HIV antibodies; respiratory viral testing: negative for influenza A/B antigens and severe acute respiratory syndrome coronavirus 2 (SARS-CoV-2) nucleic acid. Both sputum bacterial culture and blood cultures were negative. Compared with the chest CT from June 17, 2024, the scan from August 15, 2024, demonstrated new, scattered streaky and patchy opacities in the left lower lobe, suggesting the possibility of a pulmonary infection in the context of fever (Fig. 1A, B, C and D). Despite combination antimicrobial therapy targeting both conventional pathogens (intravenous piperacillin-tazobactam and moxifloxacin) and NTM (oral clofazimine, rifabutin, and isoniazid; intravenous amikacin, based on the drug susceptibility testingresults from December 2023), the patient remained febrile with recurrent fevers (Table 2). Based on clinical experience, all oral medications were discontinued, and antifungal therapy with voriconazole was initiated on August 24, 2024. Although the fever frequency and peak temperature showed slight improvement initially, the patient developed high fever again three days after this adjustment. Her oxygen saturation fluctuated between 85% and 88%. With non-invasive ventilatory support set at a fractional inspired oxygen of 40%, the patient’s oxygen saturation gradually rose to 98%. A repeat chest CT on August 26, 2024, demonstrated diffuse ground-glass opacities in both lungs (Fig. 1E and F). Repeat testing for SARS-CoV-2 nucleic acid and a comprehensive respiratory virus antibody panel were negative. Serum 1,3-β-D-glucan and galactomannan antigen testing were both negative. T-lymphocyte subset analysis revealed a total T-lymphocyte count (CD3+) of 300 cells/µL, with helper/inducer T-lymphocytes constituting 25.42% (CD3 + CD4 + percentage) and an absolute count of 132 cells/µL (CD3 + CD4+). Notably, the patient’s CD4 + T-lymphocyte count was critically low at < 200 cells/µL.Integrating these findings, significant immune impairment and concurrent PJ infection in the lungs were strongly suspected. The treatment regimen was promptly supplemented with oral TMP-SMZ (0.8 g of the trimethoprim component every 8 h). Subsequently, bedside bronchoscopy performed on August 31, 2024 revealed PJ (526 sequences) and *Mycobacterium intracellulare* (57 sequences) in BALF via targeted next-generation sequencing (tNGS), confirming PJP diagnosis. While a positive NTM culture was reported from the same specimen after one month. The treatment regimen was adjusted to combination therapy with oral TMP-SMZ (0.8 g of the trimethoprim component every 8 h), intravenous methylprednisolone (40 mg daily) and intravenous caspofungin (50 mg daily maintenance dose). Following this adjustment, the patient became afebrile within 48 h with significant symptomatic improvement, oxygen saturation was maintained at 100% with non-invasive ventilatory support at a fraction of inspired oxygen of 30%. Follow-up chest CT on September 6, 2024 demonstrated marked resolution of pulmonary infiltrates compared to prior imaging from August 26 (Fig. 1G and H). After anti-infective and other symptomatic support treatments, the patient’s condition improved considerably and she was discharged home finally on September 15, 2024, continuing oral TMP-SMZ for PJ infection and antimycobacterial therapy to manage the NTM infection. In November 25, 2024, a repeat T-lymphocyte subset analysis revealed a CD3 + T-lymphocyte count of 527 cells/µL and a CD4 + T-lymphocyte of 224 cells/µL, demonstrating an increase compared to previous levels.

## Discussion and conclusions

NTM are opportunistic pathogens primarily transmitted to humans through environmental exposure to water, soil, and aerosols, with minimal person-to-person spread [1]. Notably, NTM demonstrate significantly lower contagiousness compared to Mycobacterium tuberculosis. While NTM disease can occur at any age, individuals with underlying pulmonary conditions—including chronic obstructive pulmonary disease, bronchiectasis, cystic fibrosis, pneumoconiosis, or prior tuberculosis—as well as immunocompromised hosts exhibit substantially higher susceptibility [8, 9]. The management of NTM pulmonary disease faces significant challenges due to severely limited therapeutic options, high inherent resistance rates, and treatment durations typically exceeding 18 months. These factors collectively contribute to frequent treatment failures and recurrent infections in clinical practice [1]. Notably, MAC pulmonary disease necessitates prolonged, high-intensity multidrug regimens. Unfortunately, the substantial risk of treatment-limiting adverse effects often results in therapy discontinuation and universally poor clinical outcomes [10]. Over the protracted clinical course, the patient exhibited progressive deterioration of immune function [11].

PJP, an opportunistic infection, is commonly observed in immunocompromised individuals, particularly those with AIDS, and is known for its significant morbidity and mortality rates [12]. Literature reports indicate that non-HIV immunocompromised patients with PJP often have underlying conditions such as solid organ transplantation, hematologic diseases or malignancies, autoimmune disorders, post-chemotherapy or immunotherapy, chronic kidney disease, and recovery from COVID-19 [12, 13]. Over the past few decades, the widespread use of chemoprophylaxis and antiretroviral therapy has markedly reduced the incidence and mortality of PJP in HIV-infected patients. In contrast, the increasing use of chemotherapy, corticosteroids, and immunosuppressive agents in high-risk populations has led to a significant rise in PJP cases among non-HIV immunocompromised patients, particularly those with hematologic malignancies, solid organ tumors undergoing chemotherapy, and connective tissue diseases [14]. For moderate-to-severe or refractory PJP in non-HIV patients, the combination of TMP-SMX and caspofungin were considered as an initial regimen due to the rapid disease progression and high mortality [6]. Furthermore, studies demonstrate that early administration of corticosteroids significantly reduces mortality in non-HIV patients with an arterial oxygen tension below 60 mmHg [7]. Diagnosing PJP remains challenging due to the low pathogen burden, poor sensitivity of conventional staining methods, and the limited specificity of serum biomarkers such as 1,3-β-D-glucan [14, 15]. In this case, tNGS provided a definitive diagnosis, highlighting the utility of advanced molecular techniques in improving diagnostic accuracy. Delayed diagnosis and treatment of PJP can severely impact patient outcomes and impose a substantial economic burden on families and society. Therefore, early pathogen identification and clinical intervention are critical for reducing mortality and improving prognosis in PJP patients. This HIV-negative elderly female patient, with a history of bladder tumor surgery constituting a risk factor for PJ infection, presented with 5-year refractory NTM pulmonary disease. Persistent Mycobacterium intracellulare infection induced chronic immunosuppression culminating in CD4 + T-cell exhaustion—a process exacerbated by advanced age, malnutrition, and psychological stressors [11, 14, 16–19]. Ultimately, profound cellular immunodeficiency predisposed her to opportunistic PJP. The elusive link between chronic NTM infection and compromised immunity warrants further investigation.

In conclusion, this case demonstrated the diagnostic complexity of refractory fever in patients with NTM pulmonary disease, highlighted the critical need for clinicians to maintain a high index of suspicion for concomitant PJP in long-standing NTM pulmonary disease to prevent diagnostic oversight.

**Table 1:** The patient’s blood count results

Test	Date					
	5/15	6/17	08/15	08/26	09/11	Unit
White blood cell count	5.39	6.34	7.68	9.39	6.09	10⁹/L
Neutrophil count (absolute)	3.81	5.04	6.15	9.07	4.45	10⁹/L
Neutrophil percentage	70.80	79.40	80.20	96.60	73.00	%
Lymphocyte count (absolute)	1.03	0.67	0.68	0.23	0.77	10⁹/L
Lymphocyte percentage	19.00	10.60	8.80	2.40	12.70	%

**Table 2:** The patient’s NTM drug susceptibility testing results

Medicine	MIC (µg/ml)
Clarithromycin	> 64
Rifabutin	0.12
Moxifloxacin	4
Rifampicin)	2
TMP-SMX	4/76
Amikacin	32
Linezolid	16
Ciprofloxacin	> 8
Streptomycin	> 32
Doxycycline	> 8
Minocycline	> 8
Clofazimine	0.12

Note: trimethoprim-sulfamethoxazole (TMP-SMX), minimum 0nhibitory concentration (MIC)

## Data Availability

The raw data supporting the conclusion of this article will be made available by the corresponding author.

## Abbreviations

CT: Computed tomography
PJ: *Pneumocystis jirovecii*
BALF: Bronchoalveolar lavage fluid
TMP-SMZ: Trimethoprim-sulfamethoxazole
PJP: *Pneumocystis jirovecii* pneumonia
NTM: Nontuberculous mycobacteria
MAC: *Mycobacterium avium* complex
SARS-CoV-2: Severe acute respiratory syndrome coronavirus 2
tNGS: targeted next-generation sequencing
MIC: Minimum inhibitory concentration

## References

[1] Daley CL, Iaccarino JM, Lange C, et al. Treatment of nontuberculous mycobacterial pulmonary disease: an official ATS/ERS/ESCMID/IDSA clinical practice guideline[J]. Eur Respir J. 2020;56(1). 10.1183/13993003.00535-2020 PMC8375621 32636299

[2] Haworth CS, Banks J, Capstick T, et al. British thoracic society guideline for the management of non-tuberculous mycobacterial pulmonary disease (NTM-PD)[J]. BMJ Open Respir Res. 2017;4(1):e242. 10.1136/bmjresp-2017-000242 PMC5663249 29449949

[3] Liu C, Song Y, He W, et al. Nontuberculous mycobacteria in china: incidence and antimicrobial resistance spectrum from a nationwide survey[J]. Infect Dis Poverty. 2021;10(1):59. 33926548 10.1186/s40249-021-00844-1 PMC8082609

[4] Santos-Silva A, Pereira F, Gaio R, et al. Differential risk factors for slowly and rapidly-growing nontuberculous mycobacteria: a retrospective cross-sectional study[J]. Pulmonology. 2019;25(2):114–6. 30745244 10.1016/j.pulmoe.2018.12.003

[5] Nathani A, Tauquir A, Khan S, et al. Unusual presentation of Pneumocystis jirovecii pneumonia in an immunocompromised Host[J]. Am J Respir Crit Care Med. 2023;208(11):e44–6. 37489929 10.1164/rccm.202212-2195IM

[6] Maschmeyer G, Helweg-Larsen J, Pagano L, et al. ECIL guidelines for treatment of Pneumocystis jirovecii pneumonia in non-HIV-infected haematology patients[J]. J Antimicrob Chemother. 2016;71(9):2405–13. 27550993 10.1093/jac/dkw158

[7] Ding L, Huang H, Wang H, et al. Adjunctive corticosteroids may be associated with better outcome for non-HIV Pneumocystis pneumonia with respiratory failure: a systemic review and meta-analysis of observational studies[J]. Ann Intensive Care. 2020;10(1):34. 32198645 10.1186/s13613-020-00649-9 PMC7083987

[8] Hyung K, Kim S, Kim J, et al. Rates and risk factors of progression in patients with nontuberculous mycobacterial pulmonary disease: secondary analysis of a prospective cohort Study[J]. Chest. 2024;166(3):452–60. 38499238 10.1016/j.chest.2024.03.024

[9] Wang X, Li H, Jiang G, et al. Prevalence and drug resistance of nontuberculous mycobacteria, Northern China, 2008–2011[J]. Emerg Infect Dis. 2014;20(7):1252–3. 24959839 10.3201/eid2007.131801 PMC4073877

[10] Kim J, Choi Y, Park J, et al. Impact of treatment on Long-Term survival of patients with Mycobacterium avium complex pulmonary Disease[J]. Clin Infect Dis. 2023;77(1):120–6. 36861203 10.1093/cid/ciad108

[11] Lombardi A, Villa S, Castelli V, et al. T-Cell exhaustion in Mycobacterium tuberculosis and nontuberculous mycobacteria infection: pathophysiology and therapeutic Perspectives[J]. Microorganisms. 2021;9(12). 10.3390/microorganisms9122460 PMC8704935 34946062

[12] Apostolopoulou A, Fishman JA. The pathogenesis and diagnosis of *Pneumocystis jiroveci* Pneumonia[J]. J Fungi (Basel). 2022;8(11). 10.3390/jof8111167 PMC9696632 36354934

[13] Yang L, Xia P, Zhou Y, et al. Characteristics and risk factors for Pneumocystis jirovecii pneumonia in patients with idiopathic membranous nephropathy[J]. Eur J Clin Microbiol Infect Dis. 2021;40(11):2305–14. 34047874 10.1007/s10096-021-04227-0

[14] Tang G, Tong S, Yuan X, et al. Using routine laboratory markers and immunological indicators for predicting Pneumocystis Jiroveci pneumonia in immunocompromised Patients[J]. Front Immunol. 2021;12:652383. 33912176 10.3389/fimmu.2021.652383 PMC8071988

[15] Jin F, Liu X, Chen W, et al. High initial (1, 3) Beta-d-Glucan concentration May be a predictor of satisfactory response of c Aspofungin combined with TMP/SMZ for HIV-negative patients with moderate to severe Pneumocystis jirovecii pneumonia[J]. Int J Infect Dis. 2019;88:141–8. 31442630 10.1016/j.ijid.2019.08.015

[16] Han SA, Ko Y, Shin SJ, et al. Characteristics of circulating CD4(+) T cell subsets in patients with Mycobacterium avium complex pulmonary disease[J]. J Clin Med. 2020;9(5). 10.3390/jcm9051331 PMC7290757 32375214

[17] Saeidi A, Zandi K, Cheok YY, et al. T-Cell exhaustion in chronic infections: reversing the state of exhaustion and reinvigorating optimal protective immune Responses[J]. Front Immunol. 2018;9:2569. 30473697 10.3389/fimmu.2018.02569 PMC6237934

[18] Park H, Lee W, Choi S, et al. Modulating macrophage function to reinforce host innate resistance against Mycobacterium avium complex infection[J]. Front Immunol. 2022;13:931876. 36505429 10.3389/fimmu.2022.931876 PMC9730288

[19] Prasla Z, Sutliff RL, Sadikot RT. Macrophage signaling pathways in pulmonary nontuberculous mycobacteria Infections[J]. Am J Respir Cell Mol Biol. 2020;63(2):144–51. 32160017 10.1165/rcmb.2019-0241TR PMC7397773

